# Size of Biceps Femoris Long Head Muscle Is Related to Running Economy in Male Recreational Runners

**DOI:** 10.3390/sports13110403

**Published:** 2025-11-11

**Authors:** Srivatsav Yaddanapudi, Harshvardhan Singh, John P. McCarthy, Bradley R. Newcomer, Gary R. Hunter

**Affiliations:** 1 College of Arts and Sciences, University of Alabama at Birmingham, Birmingham, AL 35294, USA; snyaddan@uab.edu; 2 Human Performance and Nutrition Research Institute, Oklahoma State University, Stillwater, OK 74078, USA; 3 Department of Physical Therapy, University of Alabama at Birmingham, Birmingham, AL 35294, USA; 4 Department of Nutrition Sciences, University of Alabama at Birmingham, Birmingham, AL 35294, USA; bnewcomer@phikappaphi.org (B.R.N.); ghunter@uab.edu (G.R.H.)

**Keywords:** knee, gait, tendon, sports, injury

## Abstract

Although the hamstring muscles play an important role in running, very little is known about the individual contributions of each hamstring muscle (biceps femoris_long head_, biceps femoris_short head_, semitendinosus, and semimembranosus) toward running economy. As such, our study examined all the muscles in the hamstring to provide insight into which muscles contribute the most to running economy. Such information can provide insight in designing precise exercise training programs for enhancing running performance. Secondary analysis from our cross-sectional study conducted on 23 male recreational runners examined the relationships between stretch shortening cycle potentiation (via leg press throw), running net VO_2_ (inverse of running economy) (at 11.3 km/h), and maximum cross-sectional area of biceps femoris_long head_, biceps femoris_short head_, semitendinosus, and semimembranosus was assessed via magnetic resonance imaging. We obtained significant correlations between the maximum cross-sectional area of the biceps femoris_long head_ and log_10_running net VO_2_ (r = −0.52; *p* < 0.05). Our multiple regression model showed that the maximum cross-sectional area of biceps femoris_long head_ but not stretch shortening cycle potentiation predicted log_10_running net VO_2_ (r = −0.52; *p* < 0.01). We found no other relationship between any other hamstring muscles and log_10_running net VO_2_. Our findings provide preliminary evidence of the importance of the biceps femoris_long head_ toward running economy. This may be due to the preferential activation of efficient slow twitch muscle fibers of the biceps femoris_long head_. Additionally, we noted that the biceps femoris_short head_, semitendinosus, and semimembranosus muscles were not related to running economy in recreational male runners.

## 1. Introduction

Running economy or inverse of running net maximal oxygen uptake (VO_2_) is defined as the steady-state oxygen consumption in response to running at a specific speed [1]. Individuals with higher vs. lower running economy consume lower oxygen and can thus run for longer periods of time without exhaustion [1]. Indeed, running economy is a strong indicator of running performance, with prior research showing that much of the variance of running performance is explained by running economy in middle distance runners [2] and physically active men [1]. Thus, creating evidence-based precise training programs to improve running performance requires insight into running economy. It is also known that running economy is negatively related to physical/muscular fatigue [3]. This is important because physical fatigue can contribute to hamstring injuries in athletes [4,5], creating significant time loss and cost for competitive athletes [6,7]. Thus, an insight into running economy could also help with preventive health care needs of athletes, beside its potential as a prognostic and diagnostic marker of hamstring injuries [8,9].

Notably, biceps femoris (BF), semitendinosus muscles (ST), and semimembranosus (SM) muscles make up the hamstring muscle group. BF, SM, and ST comprise ~40%, ~35%, and ~25% of the hamstring muscle cross-sectional area, respectively [10]. Specifically, BF_long head_ comprises ~67% of the BF and ~27% of the total hamstring muscle volume [11]. Additionally, the BF_long head_ carries a stabilizing function of the BF and the hamstring during the late-swing phase of running [9]. Notably, BF, ST, and SM muscles display differential composition of muscle and tendon amongst them. For example, the musculotendinous unit of the BF is almost 75% muscle and 25% tendon [11,12]. While these measurements are from BF_long head_, BF_short head_ is not known to contribute to running economy due to its significantly smaller muscle and tendon size [13]. Additionally, the musculotendinous units of the ST and SM have noticeably greater tendon than the BF [14]. We know that various biomechanical factors such as tendon length, tendon thickness, and physiological factors such as muscle strength and biochemical factors such as transport pump efficiencies, myosin ATPase efficiency, and mitochondrial respiration efficiency can affect running economy [1,15,16]. In addition, differences in muscle vs. tendon composition of different hamstring muscles could uniquely influence the aforementioned biomechanical and biochemical factors and could thus differentially affect running economy. However, no such information currently exists to the best of our knowledge.

There are two well-known factors which markedly influence running economy: stretch shortening cycle potentiation (SSCP; enhancement of muscle concentric force after an eccentric preloading) and the preferential activation of slow-twitch muscle fibers (PASS) [1]. It is well-established that SSCP is important for the walking and running economy [17,18]. Furthermore, ~35% to ~50% of the energy stored during the eccentric phase is used in the following concentric phase during a stretch shortening cycle activity such as leg press throw [19]. Since SSCP is positively dictated by velocity [19], more type II than type I muscle fibers may hold a greater potential in SSCP biomechanics. Interestingly, the role of PASS in the running economy is not very clear. Indeed, type I muscle fibers, which are known to be more biochemically efficient than fast-twitch muscle fibers due to greater mitochondria, are recruited first during a strenuous task [19]. Notably, maximum muscle cross-sectional area (MCSA) is a known surrogate biomarker of muscle strength, where a higher MCSA corresponds to greater muscle strength [20]. Thus, MCSA and SSCP of a muscle could be two unique factors contributing to running economy. To the best of our knowledge, whether that is true for the muscles of the hamstrings is unknown.

We do not know whether different muscle characteristics of BF_long head_, BF_short head_, ST, and SM are differentially related with running net VO_2_. Since it is non-feasible to invasively assess BF_long head_, BF_short head_, ST, SM for their muscle size, MRI-estimated MCSA is typically used as the surrogate marker of muscle size [21]. MRI-based MCSA measurements are known to have excellent inter- and intra-rater reliability in men as well as women and thus provides validity to the muscle size estimations [22,23,24,25,26]. Knowledge on the relationships of running net VO_2_ with MCSA of BF, ST, and SM, and SSCP can provide insight into understanding running economy and potentially injury risk in athletes and non-athletes. Thus, the main purpose of our study was to determine whether running net VO_2_ was related to the MCSA of the individual hamstring muscles (BF_long head_, BF_short head_, ST, and SM) and SSCP of the lower limb in recreational runners.

## 2. Materials and Methods

### 2.1. Inclusion and Exclusion Criteria

All eligible participants had completed one or more 10 km run or marathon within the previous 6 months of their study participation. In line with the pilot nature of our study, our study population was homogenous for sex. Thus, we enrolled male-only participants aged 24–40 years. All participants were physically healthy, and without any clinical conditions/medications that could affect their participation in our study. Individuals with experience with only short-distance running were not included in our study.

### 2.2. Participant Testing and Consent

We tested our participants (n = 23) over 3 separate visits. The first visit comprised testing anthropometrics and body composition.

**Figure 1:**
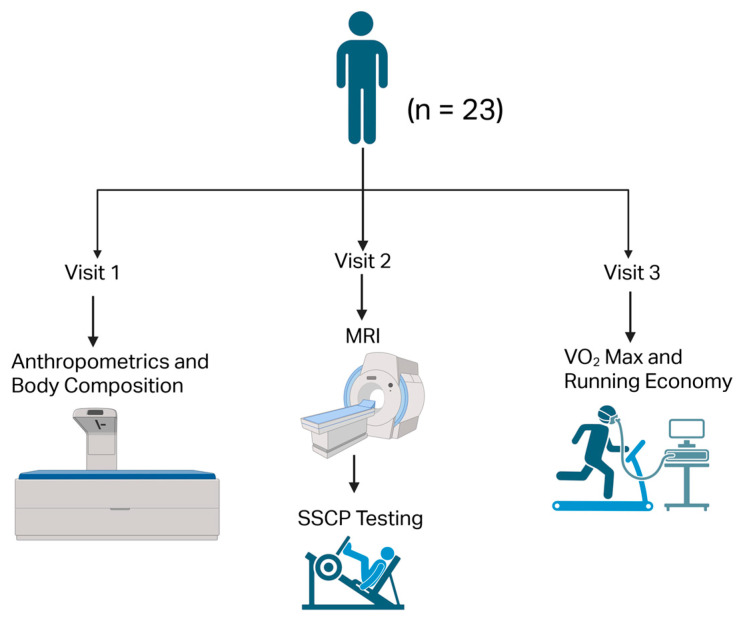
A representative image of our study design is shown above. MRI, magnetic resonance imaging; SSCP, stretch shortening cycle potentiation; VO_2_ max, maximal oxygen uptake. Created in BioRender. Yaddanapudi, S. (2025) https://BioRender.com/a65pzl2 (accessed on 10 October 2025).

SSCP and MCSA of BF_long head_, BF_short head_, ST, and SM testing occurred during the second visit. Finally, we tested VO_2_ max and running economy during the third visit. All the main outcome measures (mean ± SD) are reported in Table 1. The local Institutional Review Board approved our study. We obtained written informed consent from all the participants before any testing. The data presented in this study are secondary analysis of our previous study [19]. A representative image of our study design is shown in Figure 1. Our study was conducted in accordance with the Declaration of Helsinki [27].

### 2.3. Total Body Fat Percentage

All our participants underwent a single full-body dual-energy x-ray absorptiometry (GE Lunar Prodigy^®^, Madison, WI, USA; enCORE software, version 1.33) scan in the supine lying position to assess total body fat percentage. The coefficient of variation % for total body fat percentage is <2% in our laboratory.

### 2.4. MCSA Assessment

We used a 3-dimensional volumetric T1-weighted Turbo Field Echo imaging sequence (T1TFE) and T1-weighted Turbo Spin Echo^®^ imaging sequences (TSE) on a Philips Achieva^®^ system (3 T) (Philips Medical Systems, Best, The Netherlands) to measure the MCSA of BF_long head_, BF_short head_, ST, and SM muscles. All the scans were collected using 1H transmit/receive torso phased-array coil. Next, we obtained scout images followed by a set of unique coronal, sagittal, and axial scans from below the patient’s knee to above the subject’s thigh and below the groin. We collected all the images from the right lower extremity to stay consistent with the lower extremity used for additional muscle biopsy tests (not reported here) conducted in this study.

In total, we captured a set of 32 sagittal images (T1TFE, flip angle = 88, 32 contiguous slices, slice thickness = 2 mm, TR = 8.068 milliseconds, TE = 4.60 milliseconds, ETL = 160, acquisition matrix = 160 × 160, reconstructed matrix = 256 × 256, FOV = 250 × 250 mm) and 54 axial images (TSE, flip angle = 908, 54 contiguous slices, slice thickness = 5 mm, TR = 800 milliseconds, TE = 15 milliseconds, ETL = 3, acquisition matrix = 153 × 192, reconstructed matrix = 256 × 256, FOV = 160 × 160 mm). We used the axial images for all the MCSA measurements reported in this study.

### 2.5. SSCP Assessment

We have previously described SSCP assessment in detail [18]. First, the supine lying leg press machine (Nebula #6000-A^®^, Versailles, OH, USA) was attached with a cable and a linear position transducer (model # PT5DC-125-V62-UP-MOPO-C25; Celesco Transducer Products, Inc., Chatsworth, CA, USA) with the linear position transducer aligned to the linear motion of the leg press machine. For all the testing, the angle between the hip and back rest was set at 35 degrees with respect to the floor. Participants kept their feet 0.02m apart with the edge of their posterior heel lining with the bottom edge of the leg press foot plate for all the testing. For all the ballistic (concentric only) leg press throws, the starting position was set at 90 degrees of the knee flexion, whereas we had our participants fully extend their knees as the starting position for SSCP leg press throws. A total of 150% of the respective body weight was used for ballistic leg press throws assessment. We employed finite-difference techniques to calculate the leg press throwing velocity (33). Participants performed 3 SSC followed by 3 ballistic leg press throws with 1 min rest between each successive throw. We chose the velocity values from the best 2 trials and then averaged those for SSC throws and ballistic throws, respectively, to report in this study. The initial 10 ms difference between SSC and ballistic velocity was calculated and used as potentiated velocity metric (POTV10). As previously described [18], coefficient of variation % for POTV10 in our laboratory is <4.4% with an intraclass correlation coefficient of >0.93. Data were collected using a National Instruments (Austin, TX, USA) data acquisition system sampled at 1 kHz and digitally filtered using a low-pass 4th order Butterworth filter with a cut-off frequency of 50 Hz.

### 2.6. Running Net VO_2_ at 11.3 km/h (NVO_2_Run11)

We used a MAX-II Cart^®^ metabolic system (Physio-Dyne Instrument Company, Quogue, NY, USA) to collect seated resting and running VO_2_. Before any data collection, metabolic system calibration was conducted with a 3 L calibration syringe and known composition of standard gases. Seated resting VO_2_ was acquired while participants were seated for 10 min. Immediately after seated resting VO_2_ assessment, participants were asked to run at 11.3 km/h for 10 min. The 11.3 km/h speed was used because of its reported reliability for assessing running economy in young adult male recreational runners [19]. We subtracted the average of the last 5 min of the seated resting VO_2_ from the average VO_2_ value over the last 5 min of steady state running to obtain net running VO_2_ (NVO_2_Run11).

### 2.7. VO_2_max

After running net VO_2_ test, a VO_2_max test was conducted by having our participants run at increasing intensities for 1 min intervals starting at 9.65 km/h. Two options—increasing speed by 0.8 km/h or grade by 2.5%—were offered to our participants at the end of each successful running minute. Participants were asked to run until their maximum exhaustion. We used the following criteria to assess successful testing of VO_2_max: (a) plateauing of VO_2_, (b) respiratory exchange ratio (RER) larger than 1.2, or (c) heart rate within 10 beats of age predicted maximum.

### 2.8. Statistical Analysis

Descriptives are presented as means ± SD in Table 1. We used kurtosis, skewness, and Shapiro–Wilk tests to test the normality of our data. Using an effect size (f^2^) of 0.4, α = 0.05, power (1−) = 0.8, number of predictors = 2, and one tailed linear multiple regression fixed model with single regression coefficient model yielding a minimum of 18 participants for our study. A logarithmic conversion of the non-normally distributed NVO_2_Run11 was used for all the analyses. We used simple Pearson correlations among the variables of interest (MCSA of BF_long head_, BF_long head_, ST, SM, POTV10, and log_10_NVO_2_Run11). Finally, we used linear regression with log_10_NVO_2_Run11 as the dependent variable and POTV10, MCSA of BF_long head_, BF_long head_, ST, and SM as the independent variables. Due to technical difficulties, data were not recorded for up to 5 participants across a combination of variables as shown in Table 1. We set the level of significance for all the two-tailed analyses at *p* < 0.05. Since it is already known that SSCP is positively related to walking and running economy [28,29], we performed a 1-tailed Pearson correlation test to examine the relationship between log_10_NVO_2_Run11 vs. POTV10. All the analyses were run using SPSS software version 30.0 (SPSS Inc., IBM, Chicago, IL, USA).

## 3. Results

Our population description is provided in Table 1. Our participants, all males, ranged from 24 to 40 years with an average body mass index of ~25.2 kg/m^2^. As shown in Table 2, the MCSA of BF_long head_ and log_10_NVO2_run11_ was negatively related (r = −0.51; *p* = 0.03); however, we did not find any relationships between the MCSA of BF_short head,_ ST and SM and log_10_NVO_2_Run11 (r = 0.21, −0.34, −0.20; *p* = 0.37, 0.13, 0.37, respectively). Table 2 also demonstrates a negative relationship between POTV10 and log_10_NVO_2_Run11 (r = −0.44; *p* = 0.05). Our multiple linear regression yielded a significant negative relationship only between MCSA of BF_long head_ and log_10_NVO_2_Run11 (r^2^ = 0.31; *p* = 0.02, β − coefficient = 0.54) as shown in Table 3.

## 4. Discussion

The main finding of our study was the negative relationship between net running VO_2_ and BF_long head_ cross-sectional area. Since running economy is the inverse of the net running VO_2_, our results show that cross-sectional area of BF_long head_ is a positive contributor to running economy in male recreational runners. To the best of our knowledge, this is the first study to preliminary illustrate the importance of the MCSA of BF_long head_ toward running economy in male recreational runners. In fact, MCSA of BF_long head,_ independent of POTV10, predicted log_10_NVO_2_Run11 in our study. Importantly, we noted that BF_short head,_ ST, and SM did not play a role in running economy [19]. Since poor running economy may contribute to fatigue and fatigued muscles may increase incidence of injury, it is probable that undersized/weaker BF_long head_ fosters not only poor running economy but may also explain, in part, reduced endurance performance and increased risk of hamstring injury [4]. Thus, our preliminary findings have direct and indirect clinical implications for running performance enhancement and hamstring injury prevention.

The significance of the BF_long head_ to running economy can be attributed to the BF_long head_ role in running biomechanics [30]. The running gait cycle can be observed as follows: the stance phase, which starts at the heel strike and spans up to toe off, consisting of absorption of the eccentric forces and generation of the concentric propulsive forces which moves the body forward. On the other hand, the swing phase, which starts immediately after the toe off and finishes immediately before the heel strike, consists of mainly eccentric action of the hamstrings [30]. Specifically, the late swing phase consists of a marked eccentric force generation of the hamstring’s muscles. Since BF_long head_ is the major constituent of hamstring muscles, it is not surprising that BF_long head_ plays an important role in generating this eccentric force. A stronger and bigger BF_long head,_ meaning greater MCSA of BF_long head,_ can then contribute effectively to running economy via PASS. Indeed, a weaker and smaller BF_long head_ cannot effectively adapt to the high eccentric force demand of running which explains high incidence of hamstring injury in athletes [4].

Even though the BF_short head,_ ST, and SM constitute the hamstrings, neither the MCSA of ST or SM were related to running economy. One possible explanation for this enigma may involve the structure of the respective hamstring muscles. The ST and SM are made up of more tendon compared to muscle, with each of these muscles being up to 50% tendon [14] whereas BF_long head_ has shorter tendons and a muscle that is over twice as long as the ST and SM. Thus, it is observed that the longer BF_long head_ allowed force production over a longer period of hip extension and/or knee flexion, thus contributing more to the significant eccentric force generation and energy cost of running [11]. This is supported by our previous findings where we showed that muscle fibers operating over a smaller range are ineffective in generating a greater eccentric torque, specifically at a high velocity [19]. Furthermore, it is known that the operating range of optimal fiber length of muscles with long tendons is markedly different from muscles with shorter tendons for a functional activity such as gait or jump [19,31]. Thus, differential muscle-tendon unit of the BF_long head_ may, in part, dictate the importance of BF_long head_ MCSA of toward running economy in our study [32].

Another factor explaining the relationship of MCSA of BF_long head_ with running economy could be the ability of stronger efficient slow twitch muscle fibers of the BF_long head_ to accomplish a larger proportion of the knee flexion/hip extension that occurs during running. Indeed, preferential activation of type I muscle fibers during running has lent itself to a better running economy in a previous study [19,33]. BF_short head_ comprises ~10–12% of the hamstring muscle volume so it is not surprising that we did not find any relationship of running economy with BF_short head_. Additionally, it is known that larger muscles also consist of increased muscle fibers, with both capable of increasing with resistance training [34]. Thus, muscle length and muscle size of the BF_long head_ could be major factors explaining its significant relationship with running economy in our study.

In line with previous studies which found that increased muscle strength may allow for increased contributions of SSCP towards running economy, our results also showed the trend of positive relationship between SSCP (POTV10) and running economy at 11.8 km/h [19]. Notably, we did not find any relationship between BF_long head_ and POTV10 indicating that the contribution of BF_long head_ toward running economy is not dependent on SSCP. Interestingly, our regression result showed that only MCSA of BF_long head_ but not POTV10 predicted running economy. This was not surprising to us because of the following reasons. The measure of SSCP in our study was collected via a modified leg press device. Since the leg press movement is primarily governed by the quadriceps femoris and plantar flexors and not the hamstrings, SSCP measure in our study may not truly reflect the hamstring muscles. Moreover, the SSCP measure in our study did not come from any gait-related measures. As mentioned earlier in the discussion of the running gait cycle, hamstring muscles are activated to a greater degree between toe-off to heel-strike and a stronger hamstring muscle can result in a more efficient completion of this phase [12]. This can serve as an explanation for why only the MCSA of BF_long head_ was related to running economy. Future studies should assess SSCP of the BF_long head_ via different gait tasks. Such information then could provide mechanistic information on the underlying mechanism of the contribution of BF_long head_ toward running economy.

### Limitations

The results of our study should be taken with some limitations. Our results are preliminary in nature so external validation with larger sample size is needed to confirm our findings. Our results do not infer causation because of the cross-sectional design of our study. Furthermore, our study only assessed males so we do not know if these results could be generalized to females. Moreover, certain factors such as footwear, running technique, and running styles that were not evaluated in this study could influence our results. We do not know if any of our participants experienced any pain during the testing which might have affected their performance. However, our participants were physically fit and thus it can be surmised that pain did not affect our participants’ performance. In addition, we do not have data on fiber phenotyping of individual hamstring muscles. Such data could provide an even more detailed and informed understanding of muscle-specific role within the hamstrings toward running economy.

## 5. Conclusions

Overall, our findings provide preliminary evidence of the importance of the BF_long head_ size towards running economy in male recreational runners. Our findings also have great clinical implications. For example, MCSA of BF_long head_ can be used as a prognostic marker for running economy in athletes. Data on MCSA of BF_long head_ may also be explored to examine its interrelationships with fatigue and hamstring strain injury. Furthermore, information on MCSA of BF_long head_ can be useful for the health care practitioners to make return-to-sport decisions for athletes.

Our data needs to be replicated in different populations including athletes of various levels to confirm our current findings. Additionally, whether our findings apply to different age or sex of runners needs to be established. Our results provide a framework to investigate muscle-mechanism-specific contribution to running economy in male recreational runners. Would it hold true with female runners or older runners remain exciting areas of future determination. Overall, a greater mechanistic understanding of each muscle of the hamstrings group has the potential to inform clinical care of hamstring injuries.

## Figures and Tables

**Table 1:** Descriptive statistics.

Variables	Mean ± SD (n = 23)
Age (years)	31.73 ± 4.72
Height (m)	1.80 ± 0.08
Body mass (kg)	80.13 ± 10.79%
Body fat percentage (%)	17.90% ± 6.53%
Body mass index (kg/m^2^)	25.21 ± 3.24
MCSA BF_long head_ (mm^2^) (n = 18)	1906 ± 344.66
MCSA BF_short head_ (mm^2^) (n = 21)	802.52 ± 210.58
MCSA SM (mm^2^) (n = 21)	1567.52 ± 346.52
MCSA ST (mm^2^) (n = 21)	1109.62 ± 252.81
VO_2_ max (mL/kg/min) (n = 22)	54.51 ± 7.36
NVO_2_Run11 (mL/kg/min)	38.00 ± 4.82
Log_10_NVO_2_Run11	1.58 ± 0.05
POTV10 (m/s) (n = 21)	0.07 ± 0.06

Abbreviations: MCSA = maximum cross-sectional area; BF = biceps femoris; SM = semimembranosus; ST = semitendinosus; NVO_2_Run11 = net running VO_2_ at ~11 km/h; POTV10 = potentiated velocity at initial 10 ms of the concentric phase during a leg press throw.

**Table 2:** Correlation table.

MCSA	POTV10	LogNVO_2_Run11
MCSA SM	r = 0.07	r = −0.20
	*p* = 0.76	*p* = 0.37
MCSA ST	r = 0.17	r = −0.34
	*p* = 0.47	*p* = 0.13
MCSA BF_short head_	r = −0.02	r = −0.21
	*p* = 0.95	*p*= 0.37
MCSA BF_long head_	r = −0.07	**r = −0.56**
	*p* = 0.79	***p* = 0.03**
POTV10	r = 1	r = −0.44 *p* = 0.05 *

Abbreviations: MCSA, maximum cross-sectional area; SM, semimembranosus; ST, semitendinosus; POTV10, potentiated velocity at 10 ms into the concentric phase of the leg press throw; LogNVO_2_Run11; logarithmic converted value of net running VO_2_ at 11 km/h. * Trend toward significance. Bolded correlation between MCSA BF_long head_ and LogNVO_2_Run11 indicate statistical significance.

**Table 3:** Multiple regression model relating Log_10_NVO_2_Run11 with BF_long head_ MCSA and POTV10. Model Summary R^2^ = 0.31.

Variable	Unstandardized B	B-Coefficients	Partial r	95% CI (Lower CI–Upper CI)	Sig. (*p*-Value)
Constant	53.46			41.72–65.20	<0.001
POTV10	−17.71	−0.23	−0.27	−52.88–17.47	0.30
MCSA BF_long head_	−0.01	0.54	−0.55	−0.01–−0.001	**0.02**

Abbreviations: CI, confidence interval; POTV10, potentiated velocity at 10 ms of concentric phase of; MCSA, maximum cross-sectional area; BF, bicep femoris; β-coefficients display changes in SD in dependent variable per SD change in independent variable. Bolded *p*-value indicates statistical significance between MCSA BF_long head_ and LogNVO_2_Run11.

## Data Availability

Data are contained within the article. In addition, dataset is available on request from the authors.

## Abbreviations

The following abbreviations are used in this manuscript:

MCSA	Maximum cross-sectional area
BF	Biceps femoris
SM	Semimembranosus
ST	Semitendinosus
NVO_2_Run11	Net running VO_2_ at ~11 km/h
POTV10	Potentiated leg press throw velocity at initial 10 ms of the concentric contraction
SSCP	Stretch shortening cycle potentiation
